# Chemical tools targeting readers of lysine methylation

**DOI:** 10.1016/j.cbpa.2023.102286

**Published:** 2023-03-20

**Authors:** Gloria Ortiz, Tatiana G. Kutateladze, Danica Galonic Fujimori

**Affiliations:** 1 Department of Cellular and Molecular Pharmacology, University of California San Francisco San Francisco, CA 94158, USA; 2 Department of Pharmacology, University of Colorado School of Medicine, Aurora, CO 80045, USA; 3 Department of Pharmaceutical Chemistry, University of California San Francisco San Francisco, CA 94158, USA; 4 Quantitative Biosciences Institute (QBI), University of California San Francisco San Francisco, CA 94158, USA

**Keywords:** Reader domains, Chemical probes, Post-translational modifications, Lysine methylation, Peptidomimetic ligands, Small-molecule antagonists

## Abstract

Reader domains that recognize methylated lysine and arginine residues on histones play a role in the recruitment, stabilization, and regulation of chromatin regulatory proteins. Targeting reader proteins with small molecule and peptidomimetic inhibitors has enabled the elucidation of the structure and function of specific domains and uncovered their role in diseases. Recent progress towards chemical probes that target readers of lysine methylation, including the Royal family and plant homeodomains (PHD), is discussed here. We highlight recently developed covalent cyclic peptide inhibitors of a plant homeodomain. Additionally, inhibitors targeting previously untargeted Tudor domains and chromodomains are discussed.

## Introduction

Post-translational modifications (PTMs) to histone proteins are inserted, removed, and recognized by epigenetic proteins. These marks influence chromatin structure and function and regulate gene expression. One of the most common histone PTMs is the methylation of lysine residues, which is installed and removed by histone lysine methyltransferases and histone lysine demethylases, respectively. Proteins that “write” or “erase” methyl marks may also contain “reader” modules that play a role in recruiting effector proteins to chromatin or stabilizing histone binding, as well as regulating catalytic activities through allosteric mechanisms [1]. Histone lysines can be mono-, di-, or tri-methylated (Kme1/2/3), while arginine residues can be mono-methylated (Rme1) or di-methylated symmetrically (sRme2) or asymmetrically (aRme2). The Royal family of reader domains that recognize methylated lysines include the Tudor domains, proline-tryptophan-tryptophan-proline (PWWP) domains, chromodomains, and malignant brain tumor (MBT) domains [2]. PHD fingers are also common methyl-lysine and methyl-arginine readers, like Tudor domains [3].

While the cellular functions of some reader domains are still being elucidated, many are implicated in disease development and progression [4,5]. In order to uncover the role of these reader domains, the past decade has led to development of chemical probes for these targets, including methyl-lysine readers that have previously been considered undruggable [6–8]. Here we discuss advancements in the development of antagonists of methyl-lysine readers, with a special focus on the latest developments.

## Targeting PHD fingers

**Figure 1:**
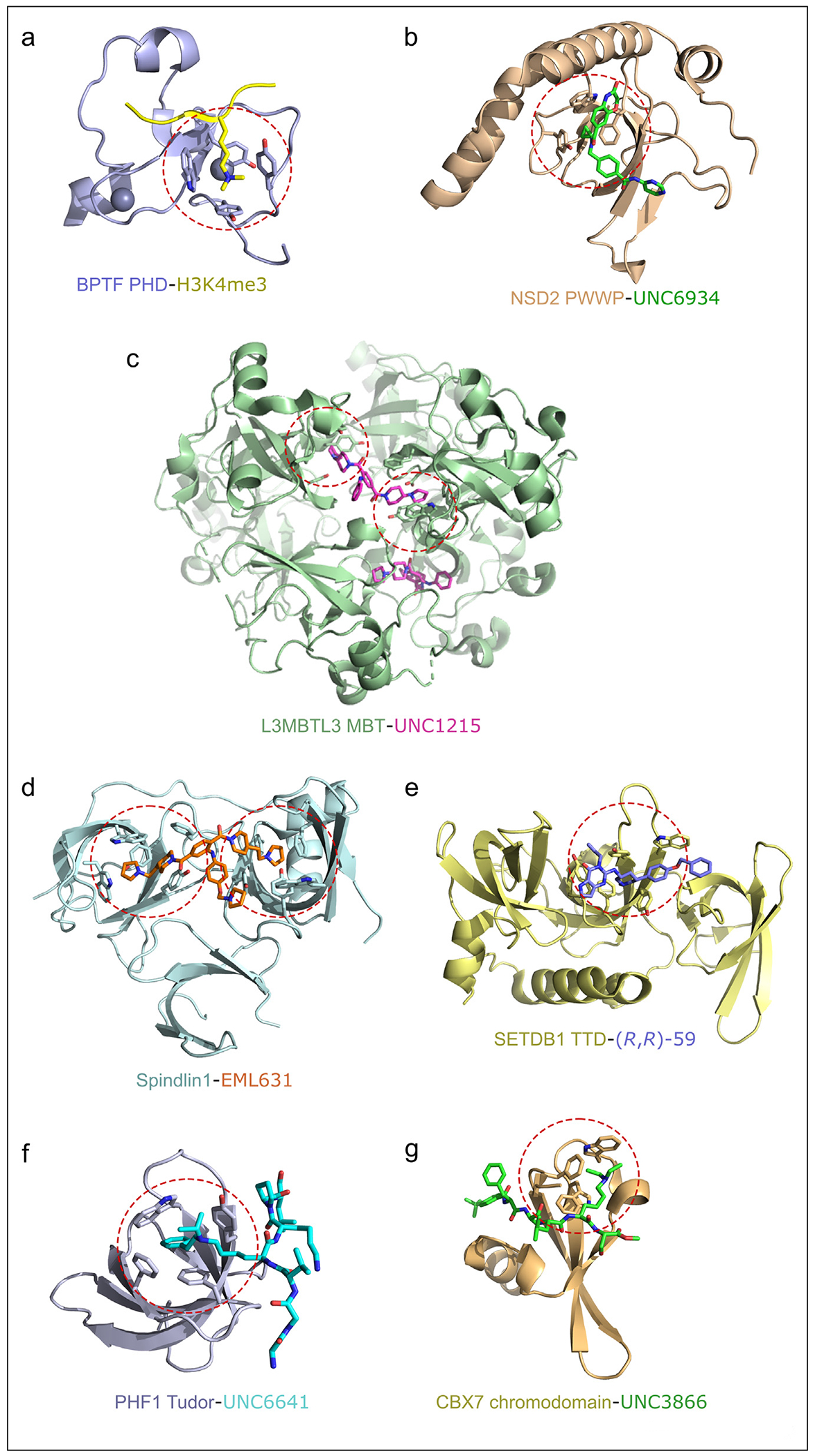
Structural mechanisms for targeting of methyllysine-recognizing readers. Ribbon representation of the structures of the readers complexes. The methyllysine-binding aromatic cages are outlined with red circles, and the aromatic residues are shown as sticks. (**a**) the PHD finger of BPTF in complex with histone H3K4me3 peptide (PDB ID: 2F6J), (**b**) the PWWP domain of NSD2 in complex with UNC6934 (PDB ID: 6XCG), (**c**) the MBT domain of L3MBTL3 in complex with UNC1215 (PDB ID: 4FL6), (**d**) Spindlin1 in complex with EML631 (PDB ID: 5JSJ), (**e**) the Tandem Tudor Doman of SETDB1 in complex with (R,R)-59 (PDB ID: 7CJT), (**f**) the Tudor domain of PHF1 in complex with UNC6641 (PDB ID: 7LKY), and (g) the chromodomain of CBX7 in complex with UNC3866 (PDB ID: 5EPJ).

**Figure 2:**
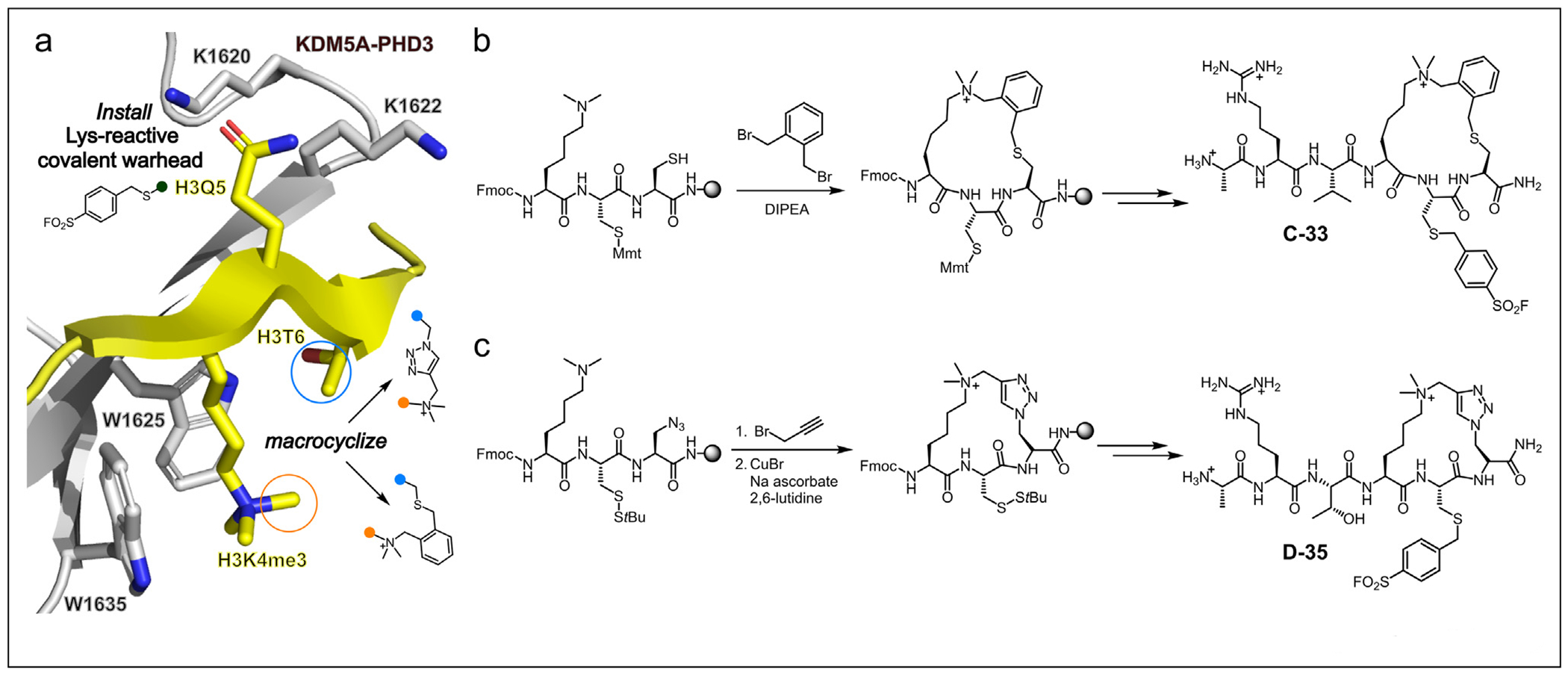
Targeting KDM5A-PHD3 with proximity-reactive cyclic peptides. (**a**) Key design principles of proximity-reactive cyclic peptides, highlighting cyclization vector and two surface exposed Lys residues present in KDM5A-PHD3 (PDB ID: 3GL6) exploited in the design of covalent inhibitors. (**b**) Thioether and triazole-forming cyclization strategies used for the synthesis of cyclic peptide PHD3 inhibitors C-33 and D-35.

One of the largest families of histone readers, PHD fingers are found in 99 human proteins [9]. PHD fingers contain 50-80 amino acid residues and are typically Cys_4_-His-Cys_3_ zinc fingers that coordinate two zinc ions. PHD fingers bind the H3 N-terminal tail, mainly recognizing unmodified H3K4 and H3K4me2/3, with a small subset recognizing unmodified H3R2, H3K9me3 and H3K14ac, with high nanomolar to low micromolar affinity [10]. Although sharing low sequence similarity, PHD fingers adopt a conserved globular structure, consisting of a two-stranded antiparallel β-sheet, variable loops connecting the zinc-binding cluster, and one or two short C-terminal α-helices [3,11,12]. Trimethylated H3K4 is recognized by an aromatic cage consisting of two to four aromatic residues and hydrophobic residues (Figures 1a and 2a) [13], while unmodified H3K4 is recognized by a combination of hydrophobic and acidic residues instead of the aromatic cage. PHD fingers have shallow binding grooves and the isolated domains are not typically considered druggable [6]. Efforts to identify inhibitors of the PHD-histone interactions have included trimethyllysine mimicking small molecules and cyclic peptides [14–16], hosts broadly targeting H3K4me3-binding or H3K9me3-binding PHD fingers [17,18], fragment-like molecules targeting the PHD fingers of BAZ2A and BAZ2B [19], and ligands for the Pygo PHD finger [20,21].

**Figure 3:**
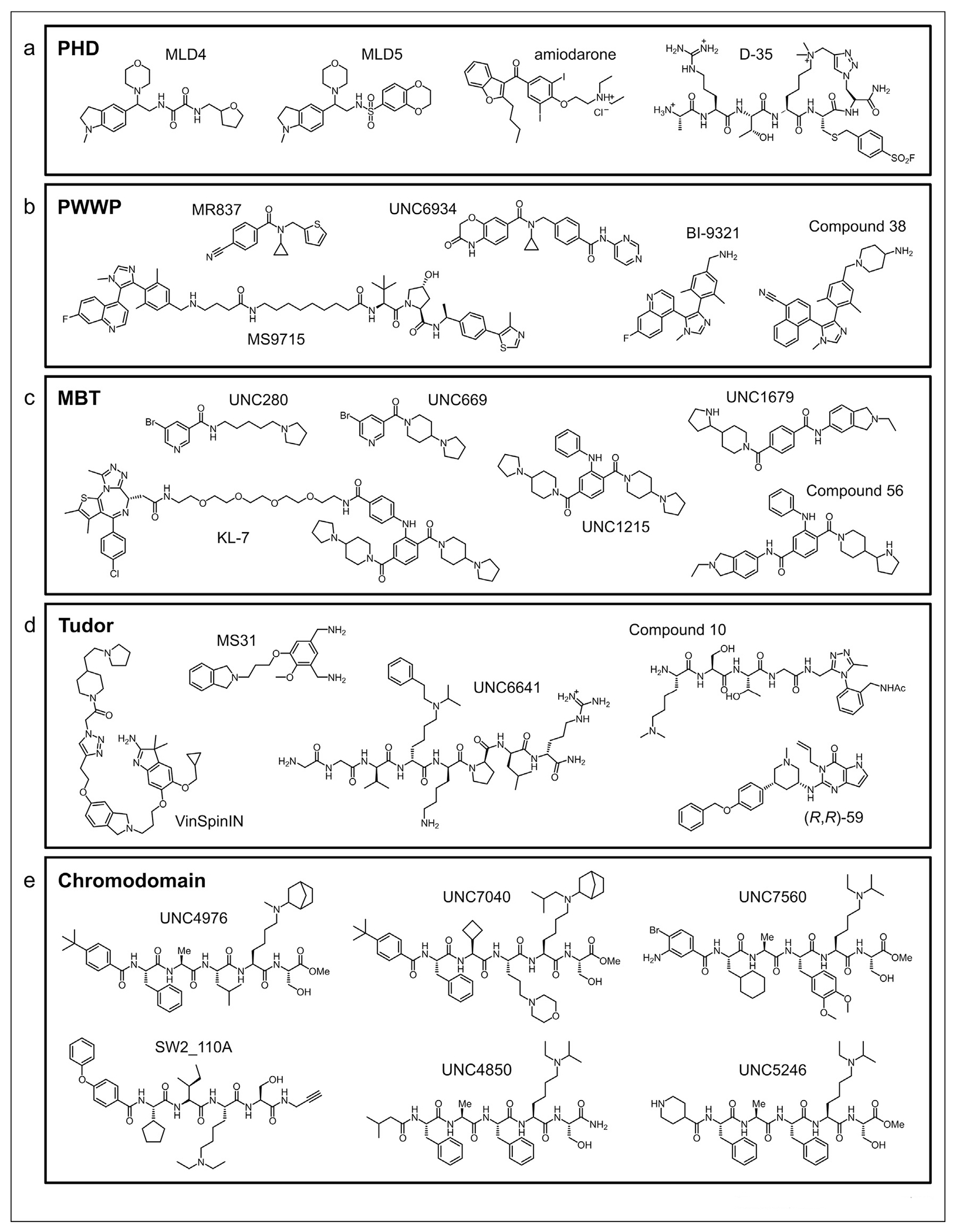
Chemical structures of histone lysine methylation reader domain antagonists. Selected ligands for (**a**) PHD fingers, (**b**) PWWP domains, (**c**) MBT domains, (**d**) Tudor domains, and (**e**) chromodomains.

In UHRF1, the PHD finger engages the H3 N-terminus and binds unmodified H3R2, while the tandem Tudor domains (TTD) simultaneously engages trimethylated K9. This bivalent and synergistic interaction is supported by the linker region that connects the TTD to the PHD finger [22,23]. Using the TTD-PHD module in an AlphaScreen-based high-throughput screen and a selectivity counter-screen, structurally similar compounds MLD3/4/5 were discovered (Figure 3a). MLD3-5 exhibited low micromolar potencies against TTD-PHD and >5-fold selectivity against other domains tested [24]. MLD3-5 displaced the full-length UHRF1 from histone peptides, specifically targeting the PHD finger of UHRF1. MLD4 and 5 decreased UHRF1-histone binding in a cellular nanoBRET assay at high concentrations (>100 μM) with no cytotoxic effects.

The PHD3 finger of KDM5A binds H3K4me3 with an aromatic groove consisting of two Trp residues, giving rise to a shallow binding surface which poses challenges for the development of small-molecule ligands. A small molecule screen focused on identifying KDM5A-PHD3 inhibitors led to weakly PHD3-binding amiodarone and several derivatives [14]. Subsequent structure-activity relationship (SAR) studies did not lead to selective inhibitors [15]. Recently, proximity-reactive cyclopeptide inhibitors of KDM5A-PHD3 were described that utilize the open binding groove to generate cyclic peptides [16]. The cyclization strategies leverage the proximity of the trimethylammonium group in Lys4 to the side chain of Thr6, while maintaining both the R2 and the tetraalkylammonium groups necessary for binding (Figures 2 and 3a). The proximity-reactive strategy takes advantage of two solvent-exposed lysine residues not present in the majority of other H4K4me3-binding PHD fingers (Figure 2a). Peptidomimetic inhibitors C-33 and D-35 contain a lysine-reactive warhead at the Q5 position and either a thioether or a triazole group tether between the quaternary K4 ε-amino group and the β-carbon of the T6 side chain (Figure 2b). D-35 shows sub-micromolar binding to PHD3, with >500-fold selectivity over the PHD1 finger of KDM5A, which binds unmodified H3K4. Stapled H3 peptides relying on separate cyclization strategies at the Thr3 and Gln5 positions, including bisthiol alkylation, were found to be more selective for the H3K4me3-recognizing BPTF-PHD and KDM4A-TTD domains, than for KDM5A-PHD3 [25]. The stapling of solvent exposed residues on the H3 peptide indicates that improved affinity and/or selectivity within H3K4me3-recognizing domains may be achieved using cyclic peptidomimetics.

## Targeting PWWP domains

Present in more than 20 human proteins, the PWWP domain is made up of 100–150 amino acid residues that form an N-terminal β-barrel core and a C-terminal α-helix bundle [26,27]. PWWP domains recognize H3K36me2/3, H3K79me3, or H4K20me3, through a typically conserved aromatic cage present in the β-barrel core, and DNA, in a flanking conversed patch of basic residues that form a positively charged surface. The first selective and potent chemical probe reported for a PWWP domain, BI-9321, was discovered through a fragment-based screening approach (Figure 3b) [28]. BI-9321 binds in the methyl-lysine pocket and displaces NSD3 from chromatin, downregulating Myc mRNA expression and reducing proliferation of leukemia cell lines. Two separate accounts report proteolysis-targeting chimera (PROTAC) molecules based on BI-9321, including MS9715, that degrade NSD3 and inhibit growth of cancer cells (Figure 3b) [29,30].

More recently, MR837 (Compound 3f), a chemical antagonist of the NSD2-PWWP1 domain, was discovered through a virtual screening campaign and several iterations of ligand-guided scaffold hopping (Figure 3b) [31]. Although a low micromolar binder, it served as the starting point for later optimization into UNC6934, a potent and selective binder of NSD2-PWWP1 (*K*_d_ = 91 nM, SPR) [32]. The cyclopropyl ring present in both MR837 and UNC6943 deeply inserts into the mildly electronegative aromatic cage pocket of NSD2-PWWP1 (Figures 1b and 3b). UNC6934 selectively binds endogenous NSD2 in live cells, disrupts H3K36me2 binding, and promotes nucleolar accumulation of NSD2. Recently described compound 38, which targets the PWWP1 domain of NSD2 (IC_50_ = 0.11 μM) and incorporates structural aspects of MR837 and BI-9321, was developed through structure-based optimization [33].

## Targeting MBT domains

The MBT domain consists of about 100 amino acid residues and is present as 2–4 repeats in 9 human proteins [34]. MBT domains recognize lower states of methylated lysine on histone and non-histone proteins and some have been identified as part of chromatinremodeling complexes [35,36]. The MBT repeats form a structural unit and only a single MBT domain contains the functional aromatic cage capable of binding methyllysine residues without significant interactions with the histone backbone. The ‘cavity insertion’ pocket typically contains three aromatic (Phe, Trp, and Tyr) residues and a conserved Asp residue that forms an ionic bond with the N–H bond in mono- and di-methyl lysine [37].

The first attempts at targeting the MBT domains of L3MBTL3 focused on virtual screening and structure-based guided peptidomimetics [38–40]. Using an AlphaScreen primary assay and an ITC counter assay [41], UNC280 and UNC669 were identified as the first low micromolar-binding, selective ligands for L3MBTL1 (Figure 3c) [40]. Subsequent SAR studies on UNC669 led to UNC926, replacing the nicotinamide with benzoic acid and displaying improved potency (~2-fold) for LMBTL1 and L3MBTL3 [42]. A target-class cross-screening approach led to the potent and selective L3MBTL3 ligand UNC1215 (*K*_d_ = 120 nM, ITC), which binds as a 2:2 complex with two separate molecules of 3MBT (Figures 1c and 3c) [43]. Later reports described UNC1679 and compound 56, which were 150-fold more and 400-more selective for L3MBTL3 than for L3MBTL1, respectively, while maintaining nanomolar affinity and cellular target engagement [44]. More recently, a targeted protein degradation strategy was developed based on the methyl reader-E3 ligase complex formed by L3MBTL3 with Cul4^DCAF5^ E3 ligase that targets methylated proteins for degradation [45]. KL-7, a PROTAC degrader based on UNC1215 coupled to JQ1, an inhibitor of BET family bromodomains, recruited the methyl reader-E3 ligase complex L3MBTL3-Cul4^DCAF5^ to BRD2, but not BRD4, for degradation in multiple cell lines (Figure 3c).

## Targeting Tudor domains

Tudor domains are found in 41 human proteins and consist of ~60 residues that make up an anti-parallel five-stranded β-barrel. Tudor domains can recognize different methylation levels of lysine and arginine residues on histone and non-histone proteins as single units or repeating units working as TTDs [46]. Spindlin1 (SPIN1) contains 3 Tudor-like domains that recognize multiple histone marks, primarily H3K4me3 via the aromatic cage of domain 2 and it can simultaneously bind H3R8me2a via domain 1 [47]. The first bivalent ligand reported for SPIN1 was EML631, a structural derivative of UNC1215 and a selective ligand (*K*_d_ = 3 μM, ITC) able to engage SPIN1 in cells (Figure 1d) [48].

A recent report describes inhibitor MS31 (*K*_d_ = 91 nM), which binds Tudor domain 2 of SPIN1 with high selectivity (Figure 3d) [49]. MS31 engages SPIN1 in cells in a nanoBRET assay with an IC_50_ of 3.2 μM. Concurrently, a bidentate ligand of SPIN1, VinSpinIN, was reported which engages Tudor domains 1 and 2 and displays high affinity (*K*_d_ < 10 nM, ITC) (Figure 3d) [50]. VinSpinIn displaced histone 3.3 from full-length SPIN1 with an EC_50_ of 270 nM in a nanoBRET assay. Selectivity profiling against reader domains and methyltransferases displayed its selectivity for Spindlin proteins.

The triple Tudor domains of SETDB1 recognizes histone tails with K14ac in a deep cavity at the interface of domains 2 and 3 and K9me1/2 via the aromatic cage of domain 3 or K9me3 via domain 2 [51]. The first report targeting SETDB1-TTD identified ligands for the Kme or Kac pocket, and compound 10, a peptide-like low millimolar-binding ligand spanning both Kme and Kac pockets (Figure 3d) [52]. More recently, (*R,R*)-59, a potent and selective small molecule inhibitor of SETDB1-TTD, was developed (Figures 1e and 3d) [53]. (*R,R*)-59 binds with nanomolar affinity (*K*_d_ = 88 nM, ITC) in the domain 2 and domain 3 region, occupying the aromatic cage of domain 2. (*R,R*)-59 inhibited histone binding to SETDB1-TTD and exhibited target engagement in cells.

The single Tudor domain in PHF1 and PHF19 recognizes H3K36me3 with micromolar affinity through the typical aromatic cage and surface–groove interactions [46]. Through mutations of 8-mer peptides and replacement of the quaternary amine in Kme3 with an aromatic (isopropyl)phenethyl-lysine residue, UNC6641 emerged as a tighter binder of the Tudor domain in PHF1 than H3K36me3 peptides, displaying a binding affinity of *K*_d_ = 0.96 μM (Figures 1f and 3d) [54]. The phenethyl group displayed additional π-interactions with the aromatic cage through π-π stacking, while the peptide formed hydrogen bonds and hydrophobic contacts along the elongated groove. UNC6641 shows >20-fold selectivity for PHF1 and PHF19 against other representative Kme readers.

## Targeting chromodomains

Chromodomains are highly conserved domains found in 29 different proteins in humans. Chromodomains consist of 40–60 residues that form a three-stranded anti-parallel β-sheet with a C-terminal α-helix. The chromodomains of the heterochromatin protein (HP1), polycomb group (PcG), and CDYL (chromodomain on the Y) families recognize the ARKS motifs in H3K9me3, H3K27me3, as well as several non-histone proteins, using an aromatic cage and surface groove interactions [55,56]. The histone peptide forms an additional β-strand, aligned anti-parallel between the first and last strands of the chromodomain.

The PcG chromobox (CBX) proteins CBX2, CBX4, CBX6, CBX7, and CBX8 are components of Polycomb repressive complex 1 (PRC1) and share a high degree of sequence homology, making targeting any individual member with inhibitors difficult. The first report targeting a chromodomain reported a 200 nM peptidomimetic antagonist of CBX4/7 with 10-fold selectivity over CBX8 [57]. Later reports utilized the first high-quality chemical probe for CBX4/7 (*K*_d_ ~ 100 nM), UNC3866 [58,59], that is > 6-fold selective over other polycomb CBXs (Figure 1g) [58,60]. Although UNC3866 displayed low cell permeability, it engaged the intact PRC1 complex in cells and inhibited proliferation of PC3 prostate cancer cells [58]. Replacing the Kme3 mimetic diethyllysine substituent in UNC3866 with the bulkier, more hydrophobic N^6^-methyl-N^6^-norbornyl present in UNC4976 gave a 14-fold increase in cellular potency for CBX4/7 and >8-fold selectivity over CBX2, CBX6, and CDYL2 (Figure 3e) [61]. UNC4976 displayed enhanced cellular activity as a positive allosteric modulator (PAM) of CBX7 chromodomain affinity for nucleic acid probes. Small molecule antagonists of CBX7 have also been described, including MS37452 (MS452), which displayed binding affinity of *K*_d_ = 29 μM and MS351, which showed weaker affinity *in vitro* (*K*_d_ = 500 μM, NMR), but higher potency in cells [62,63].

Recently, a focused DNA-encoded library (DEL) approach was used to discover a potent peptidomimetic ligand, SW2_110A, for CBX8 (*K*_d_ = 800 nM) with >5-fold selectivity over other CBX paralogs (Figure 3e) [64]. SW2_110A displayed anti-proliferative effects in MLL-AF9 leukemia cells and demonstrated that the CBX8 chromodomain is required for CBX8-mediated *HOXA9* gene activation in MLL-AF9 leukemia. The same DEL approach was used to identify selective and cell-active CBX2 inhibitor, SW2_152F [65]. SW2_152F was used to demonstrate the role of the CBX2 chromodomain in prostate cancer neuroendocrine differentiation.

More recently, UNC7040, a cellular PAM specific for CBX8 was developed guided by the available crystal structures of UNC3866 with CBX7/8 and iterative rounds of synthesis and testing (Figure 3e) [66]. UNC7040 exhibited >5-fold and >25-fold selectivity for CBX8 over CBX2/4/6 and CBX7, respectively, and an EC_50_ = 0.84 μM in a CBX8 cellular reporter assay. UNC7040 displaced CBX8-containing PRC1 from chromatin, impaired Polycomb-dependent gene silencing, and reduced proliferation of two distinct cancer cells.

Similar peptidomimetic strategies have been applied to the chromodomains of the HP1 family (CBX1/3/5), CDY family, and to MPP8, a human silencing hub (HUSH) complex member. CBX5-targeting ligand UNC7560 (*K*_d_ = 280 nM, ITC) is modestly selective for HP1 CBXs over PcG CBXs (Figure 3e) [67]. A recent study on ligand recognition by CBX5 demonstrates that larger alkyl groups at the lysine position exhibit more favorable binding, a feature that may facilitate the development of improved chemical probes for this domain [68]. In a recent study probing CDY chromodomain-histone interactions, peptidomimetic UNC4850 was identified as a sub-micromolar ligand for both CDYL2 and CDYL1b with 10-fold selectivity over CBX7 (Figure 3e) [69]. UNC4850 utilizes the same isobutyl N-terminal cap as UNC4990, a previously reported CDYL/CDYL2 ligand, to achieve this selectivity [70]. A target-class repurposing strategy was used to develop UNC5246, which binds the chromodomain of MPP8 with *K*_d_ = 0.72 μM (ITC), but also binds CDYL2 about 4-fold more potently (Figure 3e) [71].

## Conclusions

Epigenetic proteins often contain multiple reader domains that recognize multiple histone marks, either as isolated domains or in tandem. Utilizing multi-domain proteins in the discovery and/or selectivity assays may lead to more promising hits that can be developed into chemical probes and may also lead to inhibitors that target domain–domain interfaces [72]. Targeting several binding pockets present in tandem domains with bidentate ligands, like the TTD-targeted ligands VinSpinIN or (*R,R*)-59, has also been successful. Another challenging aspect of targeting reader domains are their extended, surface-exposed binding sites. PHD fingers display shallow binding grooves, suggesting that peptidomimetics may be a more tractable approach to target this class of reader domains, similar to the approach taken with chromodomain peptidic inhibitors. Despite their large size, chromodomain chemical probes retain cell permeability, a significant challenge encountered with peptidomimetics. An alternative and potentially useful strategy is to repurpose weak-binding ligands into PROTAC compounds with cellular efficacy. Based on the observation that EED-targeted PROTACs can also degrade other components of the PRC2 complex, the generality of the complex degradation by reader-directed PROTACs warrants further investigation [73,74]. Targeting readers of lysine methylation with chemical probes will aid in uncovering their functions and enable target validation.

## Data Availability

No data was used for the research described in the article.
